# Infective Endocarditis: Preliminary Results of a Cohort Study in the Southern Italian Population

**DOI:** 10.7759/cureus.8338

**Published:** 2020-05-28

**Authors:** Nicola Serra, Claudia Colomba, Paola Di Carlo, Gabriele Palermo, Teresa Fasciana, Anna Giammanco, Giuseppina Novo, Teresa Rea, Maria Michela Marino, Vincenzo Argano, Consolato Sergi

**Affiliations:** 1 Department of Molecular Medicine and Medical Biotechnology, University Federico II of Naples, Naples, ITA; 2 Department of Health Promotion, Maternal-Childhood, Internal Medicine of Excellence "G. D'Alessandro,” Promise, University of Palermo, Palermo, ITA; 3 Department of Infectious Disease, Policlinico Paolo Giaccone University Hospital, Palermo, ITA; 4 Department of Microbiology, University of Palermo, Palermo, ITA; 5 Department of Public Health, University of Naples, Naples, ITA; 6 Department of Precision Medicine, University of Campania “Luigi Vanvitelli”, Naples, ITA; 7 Cardiac Surgery Unit, University of Palermo, Palermo, ITA; 8 Medicine and Pathology: Laboratory, University of Alberta, Edmonton, CAN

**Keywords:** endocarditis, univariate analysis, matlab, gender, multi-drug resistant bacteria, microorganisms, candida endocarditis, complications, adult cardiac surgery, multivariate analysis

## Abstract

Background

Infective endocarditis (IE) is an uncommon disease with an involved interplay of clinical and surgical team management. We aimed to define diagnosis parameters and delineate in-hospital management in patients with IE admitted in a tertiary hospital of Southern Italian.

Materials and methods

Fifty-six consecutive patients (42 males, 14 females; age range: 34-85 years) admitted for IE in the Infectious Diseases, Cardiac Surgery, and Cardiology units, between January 2011 and August 2017, were enrolled. Demographic data, mortality, comorbidities, specimen type, microscopy results, special histological staining performed, and antimicrobial therapy were collected and analyzed. Any comments at the multidisciplinary team meetings were recorded in minutes of and approved.

Results

We found 83.9% of patients with positive blood cultures. The four most common bacteria were methicillin-resistant Staphylococcus aureus (MRSA: 21.3%), methicillin-sensitive Staphylococcus aureus (MSSA: 17%), Streptococci (14.9%), and Enterococci (14.9%). Both in the univariate and multivariate analysis, we observed a significant positive correlation between surgery and complications. Particularly in the univariate analysis only, surgery was positively correlated to males and C-reactive protein (CPR) at baseline. Also, considering the most common bacteria, it resulted in a positive correlation between surgery and MRSA and Streptococci spp. and between complications and MSSA. Finally, the male gender was positively correlated to MSSA and heart complications, major arterial embolism, septic pulmonary emboli, splenic infarction, and cerebral embolism.

Conclusions

A blood culture test remains a critical factor for the diagnosis of IE and the antibiotic treatment of susceptible and emerging resistant bacteria. Male gender and heart complications are red flags for prompt operative management.

## Introduction

In the last years, several efforts have been proposed to improve the management of infective endocarditis (IE) via international scientific groups [1-2]. Despite these efforts and emerging methodologies to identify the pathogens, IE remains a challenging disease with a high fatality rate. The prevalence of hospital admission for IE disease varies across European countries [3]. In the Netherlands, an increase of IE has been recorded from 30.2 new cases per 1,000,000 in 2005 to 62.9 cases per 1,000,000 in 2011 [4]. In Italy, a regional Tuscany epidemiological study over 17 years showed an incidence rate of IE of 4.6/100,000 person-years, with a higher value in people older than 65 years (11.7/100,000 person-years) [5-6]. Recently, Gouriet et al. (2018) reported a tremendous epidemiologic disparity among Mediterranean countries regarding etiology and diagnosis [7]. Blood culture is proof of continuous bacteremia and is one of the significant criteria for diagnosing IE [1-2,8-9]. Among the primary pathogens responsible for endocarditis, S. aureus is the etiologic agent most correlated to lousy prognosis [10]. There is an epidemiological trend of S. aureus endocarditis, and, in Sweden, S. aureus is now the leading cause of IE, similar to many regions of the world [10]. Apart from this, blood culture results, imaging techniques are essential for IE diagnostics because they allow diagnosis even when there are negative blood cultures, identifying the endocardial vegetation and assessing its location and relationship with the surrounding structures [11-12]. When IE is diagnosed, it is of utmost importance to understand whether the patient is at high risk of mortality or can develop severe complications, undertake first emergency procedures, and improve prognosis [13-14]. Poor prognosis is found in patients with some complications, such as neurological (middle/severe ischemic lesions or cerebral hemorrhages), cardiovascular (heart failure increases the risk of mortality twice compared to non-decompensated patients representing an indication for early surgery), and hematologic (splenic infarcts, abscesses, splenic rupture, and septic shock) [15-16]. The therapeutic approach is based on antibiotic therapy and cardiac surgery. Both procedures have a substantial impact on patient survival. Still, it is up to the “endocarditis multidisciplinary team” to understand, based on the history, local epidemiology, and clinical picture of the patient, what is the right antibiotic therapy and the timing, if necessary, of surgery [16-17]. Previously, we reported on the importance of multidisciplinary team (MDT) meetings in a Quality Assurance (QA)-certified academic setting [18].

The present study analyzed a cohort of Southern Italian patients admitted to our institution with IE, considering the demographic, clinical, and microbiological characteristics of our geographical region located in the Mediterranean basin.

## Materials and methods

This retrospective cohort study targeted 56 patients with a diagnosis of IE on discharge who were admitted to the “Paolo Giaccone” University Hospital in Palermo, Italy, between January 2011 and August 2017. Demographic data, mortality, comorbidities, specimen type, microscopy results, special histological staining performed, antimicrobial therapy were collected and analyzed by the “endocarditis multidisciplinary team.” Any comments at the MDT meetings were recorded in minutes of and approved. In our institution, the hospital team for the management of IE patients consists of cardiologists, cardiac surgeons, microbiologists, and infectious diseases (ID) physicians with close scheduled MDT meetings [19]. The study protocol conformed to the Declaration of Helsinki ethical guidelines for clinical studies and was approved by the local Ethics Committee or Institutional Research Ethics Board (REB) with approval number UIN3220. Recorded data were coded and processed, after de-identification, by one coauthor (PDC), and statistical analysis was performed by another co-author who harbors a diploma in mathematics and was not aware of the identity or results of the microbiological tests (NS).

Blood was collected according to the microbiological recommendations for the diagnosis of IE and sent to the microbiology laboratory of the Department of Sciences for Health Promotion and Mother-Child Care, where a BD Phoenix™ system (BD Diagnostic Systems, Sparks, MD) determined species identification as previously reported [1,20-21]. More than two separate blood cultures positive for coagulase-negative Staphylococci and other organisms that do not cause endocarditis were taken into account to define the microorganism responsible of IE according to the major criteria for the diagnosis of IE (Duke criteria) [2].

The identification of multidrug-resistant (MDR) gram-negative bacilli and *Candida spp. *were performed as previously reported [20-21]. Serological tests for pathogens that are epidemiologically relevant in our geographic area (i.e., *Brucella spp.*) were also performed as previously reported [22]. Prosthetic valve endocarditis was divided into early-onset (within 60 days of surgery) or late-onset (more than 60 days of operation).

Echocardiographic data consisted of a retrospective analysis of all data entered in the electronic echocardiographic database. The echocardiography laboratory team includes cardiologists with long-standing experience (more than 15 years) with the technique and with an intensive hands-on training period of the diagnosis and management of IE. According to the diagnostic algorithm proposed by the European Society of Cardiology (ESC) guidelines, all patients with a clinical suspicion of IE underwent a first complete transthoracic echocardiography (TTE) using the Siemens AcusonSC 2000 ultrasound system prime echocardiography machine (Siemens AG, Munich, Germany), equipped with a 2.5‐3.5 MHz probe within one week from the admission to the emergency unit of the University Hospital [23]. Transesophageal echocardiography (TEE) using the iE 33 echocardiographic Philips Medical System (Veenpluis, The Netherlands) was always performed in all TTE with low-quality imaging, valve prosthesis implantation, and cardiac implantable electronic devices (CIED) at the cardiosurgical unit of “Paolo Giaccone” University Hospital in Palermo as previously reported [24-26]. All echocardiographic investigations were performed according to the criteria stated in the Duke classification and discussed at the MDT meetings [2]. After discharge, patients were followed up for six months, with two-monthly outpatient interval visits.

The statistical analysis was performed using the robust Matrix Laboratory (MATLAB) analytical toolbox version 2008 (MathWorks, Natick, MA). Data are presented as number and percentage for categorical variables, and continuous data expressed as the mean ± standard deviation (SD) unless otherwise specified.

Univariate and multivariate analyses were performed. In this case, the test on Pearson’s linear correlation coefficient R was performed with the student's t-test, under the null hypothesis of Pearson’s linear correlation coefficient R = 0.

For this step, we considered the relationship between the following variables:

a) Surgery and independent variables such as age, gender, blood cultures, previous endocarditis, native valve, complications, white blood cell/red blood cell (WBC/RBC) score, C-reactive protein (CRP) values at T0 and human immunodeficiency virus (HIV) infection.

b) Complications and surgery with the independent variables represented by bacteria individuated in this study (MRSA, MSSA, Streptococci, vancomycin-susceptible Enterococcus faecalis (VSE), coagulase-negative Staphylococci (CoNS), methicillin-resistant coagulase-negative Staphylococci (MR-CoNS), multidrug-resistant (MDR) E. coli, vancomycin-resistant Enterococcus app. (VRE), *Candida albicans*, *Brucella melitensis*, *Neisseria elongata subsp. nitroreducens*, and *Propionibacterium acnes*. The "complications" variable was defined considering: heart complications (heart failure, myocarditis/pericarditis, prosthetic valve dehiscence, abscess, valve perforation, valvular aneurysm, severe arrhythmias, and conduction disorders), cerebral embolism, septic pulmonary emboli, peripheral embolism, and splenic infarction.

c) Gender with complications type (heart complications, cerebral embolism, septic pulmonary emboli, peripheral embolism, splenic infarction), and bacteria type. In this step, we considered the following variables:

· Gender: male=0 and female=1

· Native valve: no involvement=0, mitral valve=1, aortic valve=2, tricuspid valve=3

· Blood cultures: presence=1 and absence=0

· Previous endocarditis: presence=1 and absence=0

· Complications: presence=1 and absence=0

· HIV+: presence=1 and absence=0

· Surgery: Yes=1 and No=0

· Heart complications: presence=1 and absence=0

· Cerebral embolism: presence=1 and absence=0

· Septic pulmonary emboli: presence=1 and absence=0

· Peripheral embolism: presence=1 and absence=0

· Splenic infarction: presence=1 and absence=0

· Bacteria (MRSA, MSSA, Streptococci, VSE, CoNS, MR-CoNS, MDR E.coli, VRE, *Candida albicans*, *Brucella melitensis*, *Neisseria elongata subsp. nitroreducens*, and *Propionibacterium acnes*): presence=1 and absence=0

Concerning the dichotomic variables, we defined their experimental probability distributions [27]. Particularly the empirical probability distributions were computed, with cumulative frequency and the probability obtained considering patient by patient. In this way, it was possible to define for dichotomous variables the corresponding probability distributions in the correlation analysis. We remark that the present continuous variables described by experimental probability distributions provided more weight to the statistical analysis results, which is also in case of the presence of only one bacterium. Finally, all tests with a p-value of less than 0.05 were considered significant.

## Results

Table 1 shows the characteristics of enrolled patients. The table also consists of more sections. The first section includes general features, risk factors, and comorbitìdities. The second and third sections show the minor and major diagnostic criteria, respectively. The fourth section describes the complications and, finally, the last section reports indications for surgical therapy.

**Table 1:** Demographics, clinical characteristics, and complications of 56 patients with infective endocarditis Minor embolic phenomena: Roth spot, Osler node; immunologic phenomena = glomerulonephritis; §Musculoskeletal manifestations = arthralgias, arthritis, and diffuse myalgia; TTE = transthoracic echocardiography; # T0 = within the first week from hospital admission; complications = major embolic phenomena and patients with clinical heart failure and echocardiogram manifestations other than vegetation; *heart complications = patients with clinical heart failure and echocardiogram manifestations other than vegetation

Variables	Value or percentage
Age years (mean ±SD)	62.8±14.9
Male	75% (42/56)
Native valve	73.2% (41/56)
Prosthetic Valve	19.6% (11/56)
Cardiac implantable electronic devices (CIEDs)	7.1% (4/56)
A history of endocarditis	10.7% (6/56)
HIV	12.5% (7/56)
Cancer	7.1% (4/56)
Hemodialysis	8.9% (5/56)
Hospital mortality rate	37.5% (21/56)
Minor Criteria	
Fever (>38° C)	87.5% (49/56)
Immunologic phenomena and minor embolic phenomena	25.0% (14/56)
Musculoskeletal manifestations§	17.9% (10/56)
Intravenous drug users	10.7% (6/56)
Congenital heart defects	1.8% (1/56)
Predisposing heart condition or intravenous drug use	1.8% (1/56)
Major diagnostic criteria	
TTE positive for IE at T_0_	87.5% (49/56)
TEE positive for IE	58.9% (33/56)
Blood culture or serological evidence of active infection	83.9% (47/56)
Complications #	60.7% (34/56)
Heart complications *	46.4% (26/56)
Arrhythmia and conduction disorders	73.1% (19/26)
Clinical heart failure	38.5% (10/26)
Abscess	11.5% (3/26)
Prosthetic valve dehiscence	11.5% (3/26)
Valve perforation	7.7 % (2/26)
Myocarditis / Pericarditis	3.8% (1/26)
Valve aneurysm	3.8% (1/26)
Cerebral embolism	21.4% (12/56)
Septic pulmonary emboli	12.5% (7/56)
Major arterial embolism	12.5% (7/56)
Splenic infarction	10.7% (6/56)
Surgery	28.6% (16/56)
Indication for surgery	46.4% (26/56)
Persistent infection	26.9% (7/26)
Heart failure	38.5% (10/26)
Abscess	11.5% (3/26)
Prosthetic valve dehiscence	11.5% (3/26)
Valve perforation	7.7% (2/26)
Valve aneurysm	3.8% (1/26)

Table 2 consists of two sections: the laboratory parameters and microorganisms identified by blood cultures (WBC, RBC, hemoglobin (Hb), erythrocyte sedimentation rate (ESR), CRP (normal values < 5 mg/dl); T_0_= in the first three days of admission, MDR).

**Table 2:** Laboratory and microbiological isolates of 56 studied patients with infective endocarditis, 2011-2017 WBC = white blood cells; RBC = red blood cells; HB = hemoglobin; ESR = erythrocyte sedimentation rate; CRP = C-reactive protein (normal values < 5 mg/dl); T0 = in the first three days of admission; MDR = multidrug resistant

Laboratory parameters	% Patients
RBC low (< 4.0 x 10^6^/µl)	64.3% (36/56)
HB (≤10 g/dl)	62.5% (35/56)
WBC (>11 x 10^3^/µl)	44.6% (25/56)
CRP at T_0_ (≥8 mg/l)	83.9% (47/56)
ESR at T_0_ (≥20 mm/hour)	46.4% (26/56)
Microorganisms identified in patients with positive (n=47) blood cultures.	% Patients
Blood culture negative endocarditis (BCNE)	16.1% (9/56)
Methicillin-resistant Staphylococcus aureus (MRSA)	21.3% (10/47)
Methicillin-sensitive Staphylococcus aureus (MSSA)	17.0% (8/47)
Streptococcus spp.	14.9% (7/47)
Vancomycine-susceptible Enterococcus spp. (VSE)	10.6% (5/47)
Coagulase-negative Staphylococci (CoNS)	8.5% (4/47)
Methicillin-resistant coagulase-negative Staphylococci resistant (MR-CoNS)	8.5% (4/47)
MDR E. coli	6.4% (3/47)
Vancomycin-resistant Enterococcus spp. (VRE)	4.2% (2/47)
Brucella melitensis	2.1% (1/47)
Candida albicans	2.1% (1/47)
Neisseria elongata subsp. nitroreducens	2.1% (1/47)
Propionibacterium acnes	2.1% (1/47)

Table 3 shows the isolated microorganisms and the cardiac site of infection. We found IE in the mitral native valve in 19 patients (33.9%), aortic native valve in 17 patients (30.4%), and tricuspid native valve in five patients (8.3%). Eleven patients (19.6%) had a valve prosthesis implantation (prosthetic mitral valve in six and prosthetic aortic valve in five). Finally, there were four (7.1%) patients with cardiac implantable electronic devices. In the native mitral native valve, we observed more presence of Staphylococci and Streptococci. In contrast, in a native aortic valve, MRSA and MSSA isolates were more frequent in comparison to others.

**Table 3:** Valves and infecting organisms in 56 enrolled patients N.B.: Some patients had more than one valve involved. CoNS = coagulase-negative Staphylococcus; MR-CoNS = resistant to methicillin coagulase-negative Staphylococcus;* N.el. sub. Nitr. *= *Neisseria elongata subsp. nitroreducens; *MRSA:* *methicillin-resistant Staphylococcus aureus​​​​​; MSSA: methicillin-sensitive Staphylococcus aureus; VSE: vancomycin-susceptible Enterococcus faecalis​​​​​​​; VRE: vancomycin-resistant Enterococcus app.​​​​​​​; MDR: multidrug-resistant​​​​​​​

Microorganism	Mitral native valve (19 pts)	Aortic native valve (17 pts)	Tricuspid native valve (5 Pts)	Aortic prosthetic valve (5 pts)	Mitral prosthetic valve (6 pts)	Cardiac implantable electronic devices (4 pts)
CoNS		5.9% (1/17)	20% (1/5)	20% (1/5)	16.7% (1/6)	
MRSA	26.3% (5/19)	17.6% (3/17)		20% (1/5)	16.7% (1/6)	
MSSA	10.5% (2/19)	23.5% (4/17)	40% (2/5)			
MR-CoNS	5.3% (1/19)	5.9% (1/17)	20% (1/5)	20% (1/5)		
VSE	10.5% (2/19)	5.9% (1/17)			16.7% (1/6)	14.3% (1/4)
VRE				20% (1/5)		14.3% (1/4)
Streptococcus spp	21.0% (4/19)	5.9%(3/17)				
E. coli MDR					33.3% (2/6)	33.3% (1/4)
Brucella melitensis		5.9% (1/17)				
Candida albicans						14.3% (1/4)
Propionibacterium acnes					16.7% (1/6)	
N.el. sub. Nitr.	5.3% (1/19)					

Table 4 shows the univariate and multivariate linear correlation analyses between surgery and the independent variables: age, gender, blood culture, previous endocarditis, native valve, complications, WBC/RBC score, CPR at T_0_, and HIV infection. Univariate analysis showed a significant positive correlation between surgery and complications (p<0.0001) and surgery and CPR at T_0_ (p<0.0001) while there was a negative correlation between surgery and gender (p<0.0001) and HIV infection (p<0.0001). In other words, an increase or decrease of complications of CPR values at T_0_ in patients implicates an increase or decrease in the probability of surgical treatment, respectively. At the same time, male gender with IE was mostly linked to surgical therapy. The analogous response was seen for HIV infection. In multivariate analysis, we observed that HIV infection and age were negative predictors of surgery (p=0.0223, p=0.0071, respectively) while complications was a positive predictor (p=0.0212) in comparison to others. In other words, the presence of complications implicates a high probability of surgical treatment.

**Table 4:** Univariate and multivariate linear correlation analysis between surgery (dependence variable) and independence variables such as: age, gender, blood cultures, previous endocarditis, native valve, complications, WBC/RBC score, CPR at T0 and HIV+ * = significant test; R = Pearson’s linear correlation coefficient; R_partial = the partial correlation coefficient is the coefficient of correlation of the variable with the dependent variable, adjusted for the effect of the other variables in the mode; WBC = white blood cells; RBC = red blood cells; CRP = C-reactive protein (normal values < 5 mg/dl); T0 = within first week of admission; HIV: human immunodeficiency virus

Linear correlation analysis	Univariate analysis R (p-value)	Multivariate analysis (Rpartial; p-value)
		Multiple linear correlation coefficient = 0.797
Surgery / Age	-0.106 (0.438)	Rpartial = -0.392; p-value = 0.0071 *
Surgery / Gender	-0.603 (<0.0001) *	Rpartial = 0.126.; p-value = 0.405
Surgery / Blood Cultures	-0.225 (0.096)	Rpartial = 0.022; p-value = 0. 887
Surgery / Previous endocarditis	0.056 (0.684)	R partial = -0.128; p-value = 0.396
Surgery / Native valve	-0.142 (0.307)	Rpartial = 0.053; p-value = 0.726
Surgery / Complications	0.603 (< 0.0001) *	Rpartial = 0.339; p-value = 0.0212 *
Surgery / CPR at T_0_	0.642 (< 0.0001) *	Rpartial = -0.031; p-value = 0.840
Surgery / WBC/RBC score	0.213 (0.116)	Rpartial = 0.071; p-value = 0.638
Surgery / HIV infected	-0.616 (< 0.0001) *	Rpartial = -0.336; p-value = 0.0223 *

Table 5 shows the relationship between complications and surgery with isolated bacteria. It resulted that in univariate analysis, complications was positively correlated to MSSA (p<0.0001) and Brucella spp. (p=0.0106) and negatively correlated only to C. albicans (p<0.0001), i.e., presence of MSSA or Brucella spp. implicate a high probability of complications while the presence of C. albicans does not usually implicate complications.

**Table 5:** Univariate and multivariate linear correlation analyses between surgery and complications (dependence variables) with independence variables such as MRSA, MSSA, Streptococci, VSE, CoNS, MR-CoNS, MDR E. coli, VRE, Candida albicans, Neisseria elongata subsp. nitroreducens, Brucella spp., and Propionibacterium acnes * = significant test; R = Pearson’s linear correlation coefficient; R_partial = the partial correlation coefficient is the coefficient of correlation of the variable with the dependent variable, adjusted for the effect of the other variables in the mode; MRSA = methicillin-resistant Staphylococcus; MSSA = methicillin-sensitive Staphylococcus aureus; MR-CoNS = methicillin-resistant coagulase-negative Staphylococci resistant; VSE = vancomycin-susceptible Enterococcus spp; N el. Subsp. Nitr. = Neisseria elongata subsp. nitroreducens

Linear correlation analysis	Univariate analysis R (p-value)	Multivariate analysis (Rpartial; p-value)
		Multiple linear correlation coefficient = 0.983
Complications / MRSA	-0.07 (0.608)	Rpartial = -0.741; p-value < 0.0001 *
Complications / MSSA	0.54 (<0.0001) *	Rpartial = -0.555.; p-value = 0.0001 *
Complications / Streptococci	0.01 (0.934)	Rpartial = -0.663; p-value < 0.0001 *
Complications / VSE	0.04 (0.785)	Rpartial = -0.247; p-value = 0.103
Complications / CoNS	-0.04 (0.759)	Rpartial = -0.261; p-value = 0.083
Complications / MR-CoNS	0.022 (0.873)	Rpartial = -0.894; p-value < 0.0001 *
Complications / MDR E.coli	0.242 (0.072)	Rpartial = -0.339; p-value = 0.0229 *
Complications / VRE	0.022 (0.872)	Rpartial = -0.456; p-value = 0.0016*
Complications / Brucella meliensis Brucella spp.	0.339 (0.0106) *	Rpartial = -0.102; p-value = 0.505
Complications / Candida albicans	-0.727 (< 0.0001) *	Rpartial = -0.910; p-value < 0.0001 *
Complications / Neisseria elongata subsp	0.03 (0.856)	Rpartial = -0.115; p-value = 0.451
Complications / Propionibacterium acnes	0.024 (0.860)	Rpartial = -0.845; p-value < 0.0001 *
		Multiple linear correlation coefficient = 0.921
Surgery / MRSA	0.287 (0.0323) *	Rpartial = -0.210; p-value = 0.167
Surgery / MSSA	0.004 (0.977)	Rpartial = -0.403; p-value = 0.006 *
Surgery / Streptococci	0.394 (0.0027) *	Rpartial = -0.205; p-value = 0.177
Surgery / VSE	0.256 (0.057)	Rpartial = -0.03; p-value = 0.844
Surgery / CoNS	0.286 (0.0325) *	Rpartial = -0.176; p-value = 0.289
Surgery / MR-CoNS	0.034 (0.802)	Rpartial = -0.339; p-value = 0.0227 *
Surgery / MDR E.coli	-0.275 (0.0406)	Rpartial = -0.394; p-value = 0.0075 *
Surgery / VRE	0.350 (0.0083) *	Rpartial = -0.04; p-value = 0.806
Surgery / Brucella meliensis	0.285 (0.0336) *	Rpartial = 0.60; p-value < 0.0001 *
Surgery / Candida albicans	-0.674 (< 0.0001) *	Rpartial = -0.576; p-value < 0.0001 *
Surgery / N el. Subsp. Nitr.	0.10 (0.477)	Rpartial = 0.03; p-value = 0.869
Surgery / Propioni bacterium acnes	-0.119 (0.385)	Rpartial = -0.519; p-value = 0.0003 *

In multivariate analysis, we found that MRSA, MSSA, Streptococci spp., MR-CoNS, MDR E. Coli, VRE, *C. albicans*, and *Propionibacterium acnes* were all negative predictors of complications, considering all parameters simultaneously.

In univariate analysis, concerning the parameter surgery, there was a positive correlation with MRSA (p=0.0323), Streptococci spp. (p=0.0027), CoNS (p=0.0325), VRE (p=0.0083), and Brucella spp. (p=0.0336), while there was a negative correlation with C. albicans (p<0.0001) only. In other words, patients with MRSA, Streptococci spp., CoNS, VRE, and Brucella spp. had a high probability in comparison to others of surgical treatment, while *C. albicans* implicates a low probability of surgical treatment.

In multivariate analysis, MSSA, MR-CoNS, MDR *E. coli*, *C. albicans*, and *Propionibacterium acnes* were all negative predictors. At the same time, only *Brucella spp.* was a positive predictor of surgery, considering all parameters simultaneously.

In Table 6, univariate analysis shows a significant negative correlation between gender and complications type: heart complications (p<0.0001), cerebral septic embolism (p=0.0314), pulmonary emboli (0.0014), major arterial embolism (p<0.0001), and splenic infarction (p=0.0048). We found that the male gender implicates a high probability of cardiac complications, cerebral embolism, pulmonary emboli, peripheral emboli, or splenic circulatory problems.

**Table 6:** Univariate and multivariate linear correlation analysis between gender (dependence variable) with independence variables such as: MRSA, MSSA, Streptococci, VSE, CoNS, MR-CoNS, MDR E.coli, VRE, Candida albicans, Neisseria elongata subsp. Nitroreducens, Brucella spp., and Propionibacterium acnes; and gender with independence variables such as: heart complications, cerebral embolism septic, pulmonary emboli, major arterial embolism, and splenic infarction Rpartial = -0.478; p-value = 0.0009 * * = significant test; R = Pearson’s linear correlation coefficient; R_partial = the partial correlation coefficient is the coefficient of correlation of the variable with the dependent variable, adjusted for the effect of the other variables in the mode; MRSA = methicillin-resistant Staphylococcus; MSSA = methicillin-sensitive Staphylococcus aureus; MR-CoNS = methicillin-resistant coagulase-negative Staphylococci resistant; VSE = vancomycin-susceptible Enterococcus spp.; *N. el. Subsp. Nitr.* = *Neisseria elongata subsp. nitroreducens*

Linear correlation analysis	Univariate analysis R (p-value)	Multivariate analysis (Rpartial; p-value)
		Multiple linear correlation coefficient = 0.767
Gender / Heart complications	-0.674 (<0.0001) *	Rpartial = -0.448; p-value = 0.0008 *
Gender / Cerebral embolism	-0.288 (0.0314) *	Rpartial = -0.174.; p-value = 0.219
Gender / Septic pulmonary emboli	-0.416 (0.0014) *	Rpartial = -0.10; p-value = 0.497
Gender / Major arterial embolism	-0.517 (<0.0001) *	R partial = -0.419; p-value = 0.0020 *
Gender / Splenic infarction	-0.372 ( 0.0048) *	Rpartial = -0.064; p-value = 0.653
		Multiple linear correlation coefficient = 0.987
Gender / MRSA	0.033 (0.807)	Rpartial = -0.001; p-value = 0.946
Gender / MSSA	-0.422 (0.0012)*	Rpartial = -0.130; p-value = 0.394
Gender / Streptococci	-0.114 (0.404)	Rpartial = -0.025; p-value = 0.872
Gender / VSE	-0.321 (0.0159)*	Rpartial = -0.443; p-value = 0.0023 *
Gender / CoNS	-0.163 (0.231)	Rpartial = -0.11; p-value = 0.473
Gender / MR-CoNS	-0.323 (0.0152)*	Rpartial = 0.129; p-value = 0.399
Gender / MDR E.coli	-0.139 (0.308)	Rpartial = -0.559; p-value = 0.0001 *
Gender / VRE	-0.226 (0.094)	Rpartial = -0.202; p-value = 0.183
Gender / Brucella meliensis	-0.251 (0.063)	Rpartial = -0.451; p-value = 0.0019 *
Gender / Candida albicans	0.881 (< 0.0001) *	Rpartial = 0.787; p-value < 0.0001 *
Gender / N el. Subsp. Nitr.	-0.005 (0.971)	Rpartial = -0.162; p-value = 0.288
Gender / Propioni bacterium acnes	-0.129 (0.345)	

In the multivariate analysis, we found that only the cardiac complications and major arterial embolism were significant and negative predictors for gender. The presence of cardiac complications or major arterial embolism was associated with the male gender with a high probability, considering all difficulties simultaneously.

Finally, we observed in univariate analysis, a significant negative correlation between gender and bacteria: MSSA (p=0.0012), VSE (p=0.0159), and MR-CoMS (p=0.0152), while there was a significant positive correlation between gender and C. albicans (p<0.0001). In other words, the male gender was mostly associated with MSSA, VSE, and MR-CoMS, while C. albicans mainly was associated with the female gender.

In multivariate analysis, our results indicated that only *C. albicans* was a significant positive predictor for gender. At the same time, VSE, MDR *E.coli*, *Brucella spp.*, and *Propionibacterium acnes* were all negative predictors of gender, considering all parameters simultaneously. The presence of C. albicans implicates the female gender with a high probability. In contrast, VSE, MDR *E. coli*, *Brucella spp.*, and *Propionibacterium acnes* link the male gender with a high probability.

## Discussion

In our study, the diagnosis of IE was based on standard criteria, such as clinical suspicion, blood culture, and transthoracic echocardiography (TTE), and for early IE complications.

We found a high percentage of positive blood culture (83.9%), a reminder of how important it is to properly collect blood cultures and draw them before the administration of antimicrobial therapy. The percentage of positive blood culture and TTE pictures suggestive for IE, according to the Duke modified criteria, observed in our study is due to an earlier alert of an infectious disease consultant closely involved with the hospital team for the management of IE patients. In fact, in our patients, both blood culture and TTE were performed within the first days from admission to the emergency unit, confirming the utility of an early TTE [26,28-29]. Fever was the main symptom of IE, highlighting that this symptom is vital for the suspicion of IE. In our institution, the optimization of a standardized local approach to febrile patients had a crucial role in the early diagnosis of IE, and the authors support multidisciplinary teamwork, as we found it to be critical in another emergency setting. The association of fever (87.5%) with a high percentage of positive blood results (79.6%) and evidence of vegetation by echocardiography on the first days after hospital admission account for appropriate application of the Duke criteria in the diagnosis of active IE. Concerning the Duke criteria for native valve endocarditis, both sensitivity and specificity rates remain suboptimal, at nearly 80%. Electrocardiogram (ECG)-gated multidetector CT angiography (MDCTA), ECG-gated MRI, leucocyte scintigraphy, and 18F-fluorodeoxyglucose (18F-FDG) PET/CT might play a role in the diagnosis of native valve endocarditis, intracardiac prosthetic material-related infection, and extracardiac foci in adults. Notwithstanding, there is weak evidence for the diagnostic benefit of 18F-FDG PET/CT and MDCTA, such additional imaging techniques may be added in the diagnostic algorithm, if infective endocarditis is suspected. Our patients did not undergo 18F-FDG PET/CT.

Among laboratory investigations, we found a high level of CRP within three days from hospital admission confirming that high levels of CRP levels may be an inexpensive laboratory test to take to account for decision-making in febrile patients to prompt them in performing a TTE as also suggested by other studies [30]. This study analyzing the demographic variables found that age and gender influenced IE surgery therapy.

The in-hospital mortality rate during our observational period was 37.5%, with a mean age greater or equal to 63 years old. This data confirms the poor prognosis of endocarditis despite surgical treatment. The prognosis was especially poor in male patients with age of more than 63 years. Our sample showed overall complications in 60.7% of cases. These data reflect the knowledge of IE complications observed in the old and new eras [14]. Notably, the male gender was associated with cardiac and extracardiac complications. This result may be explained considering that males with IE have been reported to have a high probability of cardiac surgery as compared with females. Recent studies confirm that male gender and old age are variables that determine who may need surgery irrespective of the clinical status and comorbidities [30].

Moreover, our male patients were mostly associated with more pathogens such as MSSA, VSE, and MR-CoNS. Apart from the microorganisms identified in the cardiac valves, the exciting picture of our study is the diversified etiology identified with new organisms such as *Candida albicans*, *Brucella melitensis*, *Neisseria elongata subsp. nitroreducens*, *Propionibacterium acnes*, and multidrug-resistant gram-negative bacteria. Our study performed in a QA-certified institution may highlight that the treatment of IE is challenging now. The early identification of pathogens is the key to start appropriate antibiotic therapy, and new drug combinations may be needed in the nearest future.

Our microbiological data showed that MRSA, MSSA, and Streptococcus spp. were the most frequent isolates, while *Candida albicans*, *Brucella melitensis*, *Neisseria elongata subsp. nitroreducens*, *Propionibacterium acnes* were less persistent. Mainly three cases of MDR E. coli were observed in prosthetics and CIEDs, and they could be considered nosocomial infections. We found 15% of our patients with IE due to Enterococcus spp. and this rate is similar to the rate associated with Streptococcus spp. showing the changing epidemiological pattern. This is according to other studies that reported that Enterococcus emerged as the third most common pathogen (10%-15% of IE cases) following Staphylococci and Streptococci.

The treatment of IE due to Enterococcus spp. is challenging specifically if it is due to VRE, if it is nosocomial, and if it is associated with bacteriemia of the genito-urinary tract.

Our results showed that complications were positively correlated to MSSA. This setting is probably due to the consideration that in half of the patients with MSSA endocarditis, there is an involvement of the aortic valve. Indeed, the involvement of the aortic valve is associated with more complications as identified in the literature.

The increase in the average age of IE onset and the increase in admissions implicate an increasing rate of probability to receive antibiotic therapy, with the development of antibiotic resistance that has reached dramatic numbers in our study (almost half of the cases). In this study, we verified that isolation of S. aureus showed unfavorable prognosis, considering that they represent 18/47 isolates and that half were resistant to methicillin, prompting our team to reduce the incidence of this microorganism.

Further, we found some resistant strains of both E. coli and Enterococcus spp. This situation should be considered a red flag able to guide the clinician in the initial phases of antibiotic therapy. In our institution, we acted to improve prevention in risk categories, early diagnosis, and targeted therapy through periodic antibiograms.

The limitations of the present study are associated with the small size of the sample, but this study, unlike others, investigates many aspects related to patients with EI. In particular, it aims to represent a preliminary study on a cohort of patients to try to identify possible predictors of surgery and complications type among bacteria and clinical parameters. Also, the use of the experimental probability distributions in correlations analysis could emphasize some dependencies even with variables/parameters characterized by small absolute frequencies. It may be essential to address the sample bias using multicentric studies in the future.

## Conclusions

In conclusion, we would like to emphasize that the pattern of the etiology of IE is rapidly changing, and emerging resistant pathogens should be the goal of IE management. Multidisciplinary management and early surgical treatment are imperative in reducing mortality, identifying the principal risk factors such as male gender and preoperative cardiac complications.
